# Increase of Colon and Rectal Cancer Incidence Rates in Japan: Trends in Incidence Rates in Miyagi Prefecture, 1959-1997

**DOI:** 10.2188/jea.16.240

**Published:** 2006-11-03

**Authors:** Yuko Minami, Yoshikazu Nishino, Yoshitaka Tsubono, Ichiro Tsuji, Shigeru Hisamichi

**Affiliations:** 1 Division of Community Health, School of Health Sciences, Tohoku University Faculty of Medicine.; 2 Division of Epidemiology, Miyagi Cancer Center Research Institute.; 3 Miyagi Prefectural Cancer Registry, c/o Miyagi Cancer Society.; 4 Division of Health Policy, Tohoku University School of Public Policy.; 5 Division of Epidemiology, Department of Public Health and Forensic Medicine, Graduate School of Medicine, Tohoku University.; 6 Miyagi Cancer Center Research Institute.

**Keywords:** Models, Statistics, Cohort Effect, Colon Neoplasms, Incidence, period effect

## Abstract

**BACKGROUND:**

During recent decades, colorectal cancer incidence rates have been rapidly increasing in Japan. To investigate trends in colorectal cancer incidence rates, we analyzed incidence data during 39 years between 1959 and 1997 in Miyagi Prefecture, Japan.

**METHODS:**

Using age-period-cohort models, we evaluated the effects of time period and cohort on colon and rectal cancer incidence. Model fitting was based on eleven 5-year age groups (30-34 to 80-84), eight 5-year time periods, and 18 overlapping birth cohorts of 10 years each.

**RESULTS:**

The analysis found a significant (p=0.04) and upward period effect on female colon cancer incidence, and a significant (p<0.01) and upward cohort effect on male colon cancer incidence. An upward period effect was also observed for male colon cancer incidence without significance. For rectal cancer incidence, a significant cohort effect was found among both males and females.

**CONCLUSIONS:**

In light of known risk factors of colorectal cancer, the effects of period and cohort might be related to the change in the prevalence of risk factors such as high intake of meat and animal fat, and obesity. The improved diagnostic procedures including the spread of cancer screening might be responsible for the period effect. Although the significant cohort effects may give a caution for a continuous increase of colorectal cancer incidence, the future trend may be influenced by the period-related factors. Successive monitoring of cancer incidence and prevalence of risk factors is required.

In the mid-twentieth century, colorectal cancer incidence was low in Japan.^1^ However, since then, colorectal cancer incidence rates have been increasing rapidly.^2^ According to the latest report from the Miyagi Prefectural Cancer Registry (MPCR), the oldest cancer registry in Japan, colon cancer has become the third leading cancer site in both male and female cancer incidence, and rectal cancer has become the fourth leading cancer site in male cancer incidence.^2^ While the increase of colorectal cancer incidence has also been observed in several Asian countries,^3^^,^^4^ the rapid increase in Japan is a conspicuous phenomenon. Furthermore, compared with high risk countries such as the United States and other Western countries in which colorectal cancer incidence has been stabilizing or declining,^3^^,^^5^ Japan is now regarded to be one of the high-risk countries for colorectal cancer.^3^^,^^6^ During recent decades, lifestyles and behaviors in Japanese have largely changed. The increased colorectal cancer incidence may be related to this change of lifestyles.

In the present study, we obtained colon/rectum cancer incidence data during 39 years between 1959 and 1997 from the MPCR and investigated the trend in the incidence, using age-period-cohort models. Through statistical analysis, period and cohort effects were disentangled. In light of the known risk factors of colorectal cancer and other suspected factors, we attempted to explore factors which might be responsible for the period and cohort effects.

## METHODS

### Incidence Data

Incidence data of colon and rectal cancer were obtained from the MPCR. Miyagi Prefecture is located in the northeastern part of Japan, and its population as of 1995 was 2,328,739 (male 1,144,739, female 1,184,000). The MPCR, which was initiated in 1951 and reorganized in 1959, covers the entire prefecture. Cancer cases are registered from clinics and hospitals, radiology and pathology departments, autopsy records, mass screening records, and death certificates. Cancer incidence data since 1959 have been stored and reported.^1^

The present study observed the incidence data of colon cancer (ICD-7th; 153, ICD-8th; 153, ICD-9th; 153, and ICD-10th; C18) and rectal cancer (ICD-7th; 154, ICD-8th; 154, ICD-9th; 154, and ICD-10th; C19 - C21) between 1959 and 1997. Intramucosal cancer has been counted as an incident case. In the early period, the incidence data from the MPCR were stratified by an unequally spaced time period. Thus, the incidence data have been divided into 9 time periods (1959-61, 1962-64, 1965-67, 1968-72, 1973-77, 1978-82, 1983-87, 1988-92, and 1993-97). Based on this original incidence data, we first calculated age-specific incidence rates for 18 five-year age groups (0-4 to 85+ years) by period. As a denominator, the population at mid-year of each period was used: in census years, census population, and in non-census years, population estimated by linear interpolation using the censuses, was adopted. Furthermore, to look into the overall trend in colon/rectum cancer incidence rates, age-standardized incidence rates were calculated using the Japanese 1985 model and world populations as standards.

### Statistical Methods

To investigate the effects of period and cohort on colon/rectum cancer incidence, we used age-period-cohort models.^7^^-^^9^ The statistical method has already been described elsewhere.^10^^,^^11^ In the present study, the age-period-cohort analysis was performed for ages 30-84 according to site and sex.

In the analysis, we assumed that the interval widths for age and period were equal. Therefore, we reorganized the above original incidence data into eight 5-year time period groups including 1958: 1958-62, 1963-67, 1968-72, 1973-77, 1978-82, 1983-87, 1988-1992, and 1993-1997. In this reorganization, we estimated the number of 1958-62 incidence cases by multiplying the number of 1959-1961 (3-year period) incidence cases and 5/3. The number of 1963-67 incidence cases was estimated by multiplying the number of combined 1962-64 and 1965-67 (6-year period) incidence cases and 5/6. Thus, model fitting was based on eleven 5-year age groups (30-34 to 80-84), eight 5-year time periods (1958-62 to 1993-1997), and 18 overlapping birth cohorts of 10 years each (1873-82 to 1958-67).

A general form of the age-period-cohort model is

log(*λ*_ijk_)=*μ*+*α*_i_+*π*_j_+*γ*_k_,

where *λ*_ijk_ is the rate in a particular category, i.e., *λ*_ijk_ =d_ijk_/n_ijk_ (d_ijk_: the number of cancer cases, n_ijk_: person-years) and *α*_i_ represents age effects, *π*_j_ period effects, and *γ*_k_ cohort effects.^7^^-^^9^ To fit the model and estimate the parameters, we used the maximum likelihood method. The number of cancer cases in each category (numerator of the rate) was assumed to have a Poisson distribution, and person-years for each category (denominator) were fixed. The person-years were calculated by summing the population counts in the census year, and those in the non-census years which were estimated by linear interpolation using the censuses. The modeling procedure was performed using the GLIM system.^12^ In the GLIM program, the number of cancer cases, d_ijk_, was specified as the y-variate, Poisson errors with log link and log(n_ijk_) as an offset, and then the terms of age, period and cohort were fitted.^13^^,^^14^ Among these terms, age was entered into all models.

Usually, when a Poisson distribution is assumed for the number of cancer cases, the *χ*^2^-test is applicable for statistically testing the significance of each term. However, because there are many potential sources of variation in population-based data like ours, the variance may be considerably larger than the mean (overdispersion). In such cases, the quasi-likelihood approach was applied,^15^ and the F-value as shown below was used for testing the statistical significance of each term.^16^^-^^18^

F=(ΔG^2^/Δdf) / (G^2^/df),

where G^2^ and df are the Pearson chi-square and degree of freedom for the model, respectively, and ΔG^2^ and Δdf are the corresponding changes in the likelihood ratio statistic resulting from a parameter being dropped from the model.

The fit of different models compared with the age-model was judged based on adjusted R^2^_A_ (adj-R^2^_A_).^16^^,^^19^ This measure indicates how much of the variability is explained by factors other than age. For instance, the variability which period contributes is:

adj-R^2^_A_=1−(G^2^_A+P_/df_A+P_)/(G^2^_A_/df_A_).

Regarding the problem in interpreting parameter estimates from age-period-cohort models, it is known that the age, period and cohort are linearly dependent. Although non-linear effects can be uniquely estimated, it is not possible to disentangle the linear effects of the three terms (non-identifiability problem).^7^^-^^9^^,^^14^^,^^20^ The sum of the linear period and cohort effect is estimable in the models.^9^^,^^20^ In the present study, therefore, we estimated the period effects including a linear component, assuming the linear cohort effect to be zero, and estimated the cohort effects including a linear component, assuming the linear period effect to be zero, respectively.^7^^-^^9^

## RESULTS

### Age-standardized Incidence Rates during the 39 years

Table 1 shows trends in mid-year population by sex and trends in incidence counts and age-standardized incidence rates for all ages according to site and sex. Age-standardized incidence rates, which were calculated using different standard populations, showed a similar trend. Age-standardized incidence rates of colon and rectal cancer have been continuously increasing. The increase of incidence rates is more pronounced among males than females in both sites. Furthermore, compared with rectal cancer incidence, the rate of increase was more rapid in colon cancer incidence. During the observation period, the age-standardized incidence rate standardized to the Japanese 1985 model population increased 9.4 times for male colon, 4.7 times female colon, 3.9 times for male rectum, and 1.9 times for female rectum.

**Table 1.** Trends in age-standardized incidence rates of colon and rectal cancer during 1959-1997 in Miyagi Prefecture, Japan.

	Year of diagnosis								

	1959-61	1962-64	1965-67	1968-72	1973-77	1978-82	1983-87	1988-92	1993-97
Males									
Population*	848,579	846,000	858,000	889,036	960,245	1025,903	1,071,741	1,105,103	1,144,739
Colon Cancer									
Cases^†^	71	88	110	226	400	619	1,118	1,904	3,108
ASR(J)^‡^	5.1	5.7	6.3	7.8	11.9	15.8	24.3	34.4	47.9
ASR(W)^§^	3.9	4.1	4.7	5.5	8.3	11.0	17.1	24.8	34.6
Rectal Cancer									
Cases	103	102	152	279	437	603	830	1,276	1,815
ASR(J)	7.2	6.7	9.4	9.7	13.4	15.2	17.7	23.0	28.1
ASR(W)	5.3	4.8	7.0	6.9	9.2	10.9	12.8	16.6	20.5

Females									
Population	894,616	894,000	902,000	930,187	995,022	1,056,417	1,104,554	1,143,455	1,184,000
Colon Cancer									
Cases	92	102	134	279	440	705	1,101	1,574	2,273
ASR(J)	5.8	5.7	6.8	7.9	10.4	14.0	18.5	22.2	27.5
ASR(W)	4.1	4.0	4.9	5.6	7.3	10.0	13.2	15.7	19.6
Rectal Cancer									
Cases	118	126	135	269	388	557	699	862	1,075
ASR(J)	7.0	7.0	6.8	7.4	9.1	11.0	11.9	12.5	13.4
ASR(W)	4.9	5.0	5.0	5.3	6.5	8.1	8.6	9.0	9.7

* : Mid-year population. The populations in 1963 and 1966 were estimated by linear interpolation using the censuses.

† : Total incidence counts during each period.

‡ : ASR(J): Age-standardized incidence rate per 100,000 (direct age-standardization to the Japanese 1985 model population).

§ : ASR(W): Age-standardized incidence rate per 100,000 (direct age-standardization to the world population).

### Age-Period-Cohort Models

We compared age-period and age-cohort models with an age-period-cohort model, respectively, and evaluated the period and cohort effects. The model fit is shown in Table 2. In each model, age was significant.

**Table 2.** Summary statistics for age-period-cohort models of colon and rectal cancer, year 1958-1997*, aged 30-84 years.

Terms in model	Residual			Change^†^		p value for test statistics^‡^	adj-R^2^_A_
	
	Df	G^2^		Δdf	ΔG2		
			Colon				
Males							
Age (p=0.00001)	77	3385.50					
Age+period (AP)	70	180.69		16	100.30	0.00004^§^	0.94
Age+cohort (AC)	60	95.07		6	14.66	0.15404^∥^	0.96
Age+period+cohort (APC)	54	80.41					0.97

Females							
Age (p=0.00001)	77	1507.20					
Age+period (AP)	70	71.34		16	21.30	0.16727^§^	0.95
Age+cohort (AC)	60	63.49		6	13.45	0.03642^∥^	0.95
Age+period+cohort (APC)	54	50.04					0.95

			Rectum				

Males							
Age (p=0.00001)	77	1131.80					
Age+period (AP)	70	133.34		16	54.28	0.01116^§^	0.87
Age+cohort (AC)	60	84.28		6	5.22	0.73397^∥^	0.90
Age+period+cohort (APC)	54	79.06					0.90

Females							
Age (p=0.00001)	77	299.48					
Age+period (AP)	70	87.46		16	31.09	0.01310^§^	0.68
Age+cohort (AC)	60	63.49		6	11.32	0.07898^∥^	0.71
Age+period+cohort (APC)	54	56.37					0.73

* : Original incidence data for the 9 unequally spaced time periods during 1959-1997 was reorganized into 8 five-year time period groups including 1958. The method for editing this data is described in METHODS.

† : Comparisons between submodels and APC model.

‡ : For males, the F-test was used, and for females, the *χ*^2^-test was used.

§ : AP vs. APC.

∥ : AC vs. APC.

**Figure 1.**
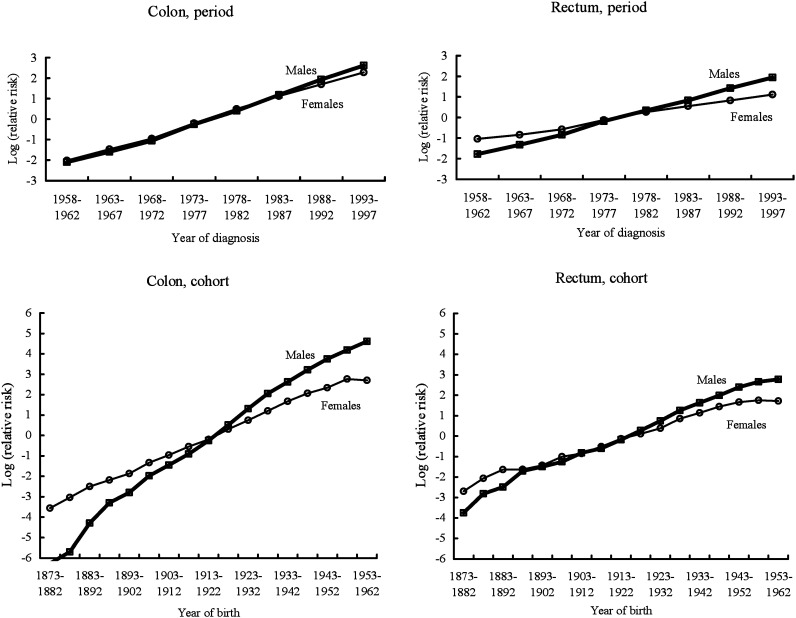
Effects of period and cohort on colon and rectal cancer incidence, based on age-period-cohort models including a linear component.

For male colon cancer incidence, the value of adj-R^2^_A_ in the full model, i.e., the age-period-cohort model, was larger than submodels. In all models, the G^2^-statistic was significant. Thus, the effect of each term was evaluated based on the F-test. The addition of the cohort to an age-period model was highly statistically significant (p<0.01), indicating the cohort effect might be significant for male colon cancer incidence. For female colon cancer incidence, the values of adj-R^2^_A_ were constant across the submodels and full model. The G^2^-statistic was not significant for all models, indicating that the *χ*^2^-test was applicable. The *χ*^2^-test shows that addition of the period to an age-cohort model gave significance (p=0.04), indicating that the period effect might be associated with female colon cancer incidence. Based on both the value of adj-R^2^_A_ in each model and the result of the statistical test for each term in males and females, we decided to use the full model for summary description in colon cancer incidence. Figure 1 (left side) shows the effects of period and cohort for colon cancer that were estimated by the full model including a linear component. In the figure, the relative risk for the last cohort 1958-67 was not plotted; because the incidence rate in the last cohort is low and the cohort contains only one cell, the risk estimate for the last cohort is uncertain. The relative risks by period and cohort increased for both males and females, respectively. A slope in the period effect was similar between males and females. In the cohort effect, the slope among males was steeper than among females.

For rectal cancer incidence, statistical testing revealed that the effect of cohort was significant among both males and females. A marginal result in the effect of period was observed for female rectal cancer incidence. In rectal cancer incidence, we also adopted the full model for summary description. Figure 1 (right side) shows the effects of period and cohort for rectal cancer which were estimated by the full model including a linear component. For the same reason as for colon cancer incidence, the relative risk for the last cohort was not plotted in the figure. An upward trend in cohort effect was observed for both male and female rectal cancer incidence. However, compared with colon cancer, the slope in the cohort effect was small. Among female younger cohorts, stabilization of the cohort effect was also observed.

## DISCUSSION

The observation of age-standardized incidence rates and the age-period-cohort analysis of the 1959-1997 data from the MPCR revealed different characteristics of time trends in incidence rates between colon and rectal cancer and between the sexes. For colon cancer incidence, a significant effect of cohort was observed among males. A slope in the cohort effect among males was steeper than among females. A significant upward period effect on colon cancer incidence was observed among females. A similar upward slope in period effect was also observed among males without significance. Thus, based on the results for colon cancer, the recent rapid increase in male colon cancer incidence rates is explained by the upward trend in the cohort and period effect, and the increase in female colon cancer incidence rates is mainly explained by the upward trend in the period effect. On the other hand, the analysis of rectal cancer incidence indicated that the increase in male and female rectal cancer incidence may be related to the upward trend in the cohort effect, although the slope in the cohort effect is not so steep compared with colon cancer.

Epidemiologic studies of colorectal cancer have identified several risk factors.^6^^,^^21^^-^^23^ Our present findings on period and cohort effects may be due to changes in the prevalence of these risk factors. However, other factors, i.e., the quality of cancer registration, the improvement of diagnostic procedures, and the spread of cancer screening, which is likely to be related to period effects, may impact the trend in incidence.^4^^,^^9^^,^^24^ It is also important to evaluate the effects of these period-related factors. First, we considered the effects of the quality of cancer registration on the trend in incidence. The quality of registration is usually evaluated by two indicators: the percentages of cases registered from death certificate only (DCO) and histopathologically verified (HV) cases. During the original 9 time periods, the percentage of DCO cases was between 7.9% (1988-1992) and 31.3% (1968-1972) for colon cancer and between 5.6% (1988-1992) and 24.1% (1968-1972) for rectal cancer, and the percentage of HV cases has been increasing (colon 1959, 39.9%, 1997, 82.8%; rectum 1959, 33.9%, 1997; 85.3%). Although the percentage of DCO cases in the MPCR is relatively high, compared with other developed countries, this percentage has been decreasing in recent decades. The increased percentage of HV cases shows that the accuracy of diagnostic information becomes higher. Thus, it is possible that the improvement of the quality of registration might have some effects on the increasing incidence, although we cannot quantify the magnitude of these effects.

Second, we considered the effects of the improvement of diagnostic procedures and the spread of cancer screening on the trend in incidence. Previous studies have suggested that the improvements of diagnostic procedures and the spread of cancer screening have some effects on colorectal cancer incidence.^5^^,^^25^ The use of colonoscopy and sigmoidoscopy in screening may reduce the risk of colorectal cancer through endoscopic removal of precancerous lesions, or, on the contrary, may produce an increase in colorectal cancer incidence through early detection of cancer. In Miyagi Prefecture, the proportion of screen-detected colon/rectum cancer cases has been increasing since colorectal cancer screening was introduced as a public health program in 1993 (Table 3).^26^ The proportion of screen-detected cancer in colon was slightly higher than that in rectum (Table 3). Furthermore, as shown in Table 1, the age-standardized colon cancer incidence rate in 1993-1997 standardized to the Japanese 1985 model population has increased 1.4 times for males and 1.2 times for females, compared with the incidence in 1988-1992. Although no data on trend according to clinical stage is available, early-stage and intramucosal cancer cases may be increasing. Thus, it is likely that the spread of cancer screening might be responsible for the rapid increase of colorectal cancer incidence, especially the increase of colon cancer incidence in Miyagi Prefecture. Although the major factor responsible for declining colorectal cancer in the United States is regarded to be the increased use of colonoscopic polypectomy for precancerous lesions,^5^ the prophylactic role of polypectomy in colorectal cancer incidence remains unclear in Japan.

**Table 3.** Trends in participation rates in colorectal cancer screening program in Miyagi Prefecture and proportions of screen-detected incidence cases in Miyagi Prefectural Cancer Registry.

	Calendar year									

	1980	1985	1990	1991	1992	1993	1994	1995	1996	1997
Participation rate in population-based screening program (%)*										
	–	–	–	–	–	19.2	16.8	19.6	19.4	20.0

Proportion of screen-detected incidence cases (%)										
Colon Cancer										
Males	3.1	4.2	9.3	11.5	13.2	25.0	29.1	26.6	25.8	25.8
Females	0.7	2.4	4.5	6.2	8.3	13.5	19.1	17.4	19.1	18.5

Rectal Cancer										
Males	1.6	5.1	6.9	6.2	8.8	21.4	23.0	22.1	23.9	20.4
Females	0.0	4.2	3.2	6.0	2.2	14.0	18.4	15.6	11.5	15.5

* : Colorectal cancer screening was introduced as a public health program in 1993.^26^ Data according to sex are not available.

As mentioned above, although the quality of cancer registration and the improvement of diagnostic procedures proved to be important period-related factors, it is unlikely that the long-term trend in colorectal cancer incidence is completely explained only by these factors. Based on previous epidemiologic studies of colorectal cancer, we further explored factors responsible for the period and cohort effects on colon/rectum cancer incidence. In Japan, Ogimoto et al^23^ reviewed risk factors for colorectal cancer. Risk factors for colorectal cancer include dietary factors such as meat and animal fat intake, physical activity, and anthropometric measures such as obesity.^6^^,^^22^^,^^23^^,^^27^

First, we compared the trend in dietary factors with the trend in colon/rectum cancer incidence. Table 4 shows the dietary data in Japan from published reports.^28^^-^^31^ Although dietary data for Miyagi Prefecture is not available, several reports indicate that recent trends in national data shown in Table 4 are similar to those around Miyagi (the northeastern part of Japan).^29^^,^^30^ During the past several decades, intake of meat, protein, fat, and animal fat has considerably increased, which may be responsible for the increased colorectal cancer incidence. Although we cannot precisely evaluate differential associations of dietary intake with the period and cohort effects, we consider that the rapid increase of meat and animal fat intake may be related to the upward period effect on colon cancer incidence. This is supported by previous findings as follows: first, the studies comparing risk factors for colon cancer and rectal cancer showed that dietary factors might be associated with colon cancer risk;^22^^,^^32^ second, some migrant studies showed that colon cancer risk increased among migrants from Japan to the US.^33^^,^^34^ Shimizu et al,^34^ for example, revealed that the colon cancer incidence among late Japanese immigrants to the US exceeded that among the homeland population, indicating that adult environments may have strong effects on the development of colon cancer. Although the migrant study provided no data on lifestyle changes in relation to age at immigration to the US, the dietary pattern in Japanese migrants might have been changed from low-fat and low-meat Japanese diet into high-fat and high-meat Western diet.^35^ Thus, based on the migrant studies, it is likely that colon cancer risk in Japanese may be easily modified by the change of some adult environments, e.g., the change of dietary pattern. In addition, a laboratory study suggests that Japanese may have a different genetic predisposition to colon cancer relative to Caucasians when Japanese are exposed to the Western diet.^36^ Rapid increase of meat and animal fat intake in Japan during recent decades, which may promote colon cancer development in the late stage, might have produced the period effect of colon cancer.

**Table 4.** Trends in dietary intake and anthropometric measures among Japanese population.

	Calendar year									

	1950	1955	1960	1965	1970	1975	1980	1985	1990	1995
Nutrient intake per capita per day*										
Energy (Kcal)	2098	2104	2096	2184	2210	2226	2119	2088	2026	2042
Meat (g)	8.4	12.0	18.7	29.5	42.5	64.2	67.9	71.7	71.2	82.3
Total protein (g)	68.0	69.7	69.7	71.3	77.6	81.0	78.7	79.0	78.7	81.5
Animal protein (g)	17.0	22.3	24.7	28.5	34.2	38.9	39.2	40.1	41.4	44.4
Fat (g)	18.0	20.3	24.7	36.0	46.5	55.2	55.6	56.9	56.9	59.9
Animal fat (g)	–	6.5	8.6	14.3	20.9	26.9	26.9	27.6	27.5	29.8
Carbohydrate (g)	418.0	411.0	398.8	384.2	368.3	335.0	309.0	298.0	287.0	280.0

BMI (kg/m^2^) by age (year)^†^										
Males										
40-49	21.5	21.5	21.9	22.0	22.3	22.9	23.1	23.1	23.4	23.6
50-59	21.3	21.4	21.7	22.0	22.2	22.3	22.6	22.8	23.3	23.4
60-69	21.0	21.0	21.4	21.4	21.7	22.0	22.1	22.6	22.6	23.2

Females										
40-49	22.1	22.1	22.6	22.6	23.0	23.1	23.2	23.1	22.8	22.8
50-59	21.9	22.0	22.3	22.7	23.1	23.4	23.3	23.5	23.4	23.3
60-69	21.5	21.5	22.1	22.4	22.6	22.9	23.0	23.5	23.5	23.6

Year of birth^§^										
	
	1931	1935	1940	1945	1950	1955	1960	1965		
		
Mean height at age 17 (cm)^‡^										
Males	160.6	162.6	163.9	165.6	167.2	168.3	169.1	170.1		
Females	152.1	152.8	153.3	154.0	155.2	155.8	156.6	157.3		

* : Data from the annual report of the cross-sectional national survey.^30^ Nutrient intake for 1950 is estimated by the Standard Table of Food Composition in Japan, 1st edition, for 1955-1960 by the 2nd edition, for 1965-1970 by the 3rd edition and for 1975-1995 by the 4th edition, respectively. Data according to sex and area are not available.

† : BMI was calculated based on mean height and mean body weight obtained from the annual report of the cross-sectional national survey.^28^^-^^30^ Data according to area are not available.

‡ : Data from the annual report of the school health statistics.^31^

§ : Year of birth was estimated from calendar year at the school health survey.

Additionally, the drastic change in nutrient intake may influence anthropometric measures, which may in turn affect cancer risk. The anthropometric data are shown in Table 4, along with dietary data. The mean of the obesity index, i.e., body mass index (BMI), has been increasing among middle- or older-aged men and women. Higher intake of energy or fat and, perhaps, sedentary lifestyles might have lead to the increase of obese men and women who are at higher risk of colon cancer. Recently, the association of obesity with colon cancer risk was reconfirmed by some cohort studies including the study in Miyagi Prefecture.^37^^-^^39^ Thus, the increasing proportion of the obese men and women in the population might also have contributed to the upward period effect on colon cancer incidence.

The risk factors may be related to not only the period effects on colon cancer incidence but also cohort effects on colon/rectum cancer incidence. However, it seems difficult to explain the cohort effects based on the known risk factors, although the cohort effect is an important determinant of trends. One possibility is that the rapid increase of height, as shown in the lower part of Table 4, might have promoted the cohort effect on male colon cancer incidence. This possibility is supported by a recent cohort study in Japan showing a significant association of height with colon cancer risk among males.^37^ In spite of unpublished data, the same association was observed in the cohort study conducted in Miyagi Prefecture. Diet during early life affecting height may also contribute to the cohort effect. However, the associations of height with colon cancer risk have not been established.^6^ We consider that the relationship between the increase of height and the cohort effect on colon cancer incidence is still hypothetical. On the other hand, as for rectal cancer, information on risk factors is less than for colon cancer.^22^^,^^32^ The association of height with rectal cancer risk was uncertain in the cohort studies in Japan. Furthermore, stabilization of the cohort effect was observed among younger female cohorts. Finally, we could not find factors which are likely related to the cohort effect on rectal cancer incidence. Additionally, in the present study, although some differences in the results of statistical testing between males and females were observed, we could not fully discuss the effect of sex on the trend in cancer incidence. This may be due to insufficient information for sex-specific risk factors and the lack of sex-specific lifestyle data.

Recently, two studies investigating the trend in colorectal cancer incidence in Japan provided conflicting conclusions.^40^^,^^41^ One noted that colon cancer incidence seems to have declined in very recent years, especially among men.^40^ The other indicates that colorectal cancer will become a major source of morbidity in Japan, as young people age and their risks increase.^41^ However, based on the present findings, we propose that it is difficult to precisely predict future trends of colon and rectal cancer incidence. Although the significant cohort effects on colon and rectal cancer incidence found in our study may give a caution for a continuous increase of colorectal cancer incidence in Japan, colon cancer risk in Japanese may be modified by the period-related factors. It is possible that colon cancer incidence may be largely influenced by the change of adult environments including dietary habits. Prophylactic roles of polypectomy in colorectal cancer incidence may emerge in the near future. Stabilization of the cohort effect on rectal cancer incidence among young female cohorts may suggest a decreasing trend in female rectal cancer incidence in future. To clarify associations of the period- and cohort-related factors with subsequent trends in colorectal cancer incidence, successive monitoring is required.

In conclusion, the present age-period-cohort analysis revealed an upward period effect on male and female colon cancer incidence, an upward cohort effect on male colon cancer incidence, and an upward cohort effect on male and female rectal cancer incidence. These effects of period and cohort might be related to the change in prevalence of risk factors and improved diagnostic procedures. Although the cohort effects on colon and rectal cancer incidence suggest a continuous increase of colorectal cancer incidence in Japan, a future trend may be influenced by the period-related factors. Successive monitoring of cancer incidence and prevalence of risk factors is required.
